# Psychological Response to a False Positive Ovarian Cancer Screening Test Result: Distinct Distress Trajectories and Their Associated Characteristics

**DOI:** 10.3390/diagnostics9040128

**Published:** 2019-09-25

**Authors:** Amanda T. Wiggins, Edward J. Pavlik, Michael A. Andrykowski

**Affiliations:** 1 Nursing Instruction, University of Kentucky College of Nursing, Lexington, KY 40536, USA; 2 Department of Obstetrics and Gynecology, University of Kentucky College of Medicine, Lexington, KY 40506, USA; 3 Department of Behavioral Science, University of Kentucky College of Medicine, Lexington, KY 40536, USA

**Keywords:** screening, ovarian cancer, distress, adjustment, trajectory analysis

## Abstract

Routine screening for ovarian cancer (OC) can yield an abnormal result later deemed benign. Such false positive (FP) results have been shown to trigger distress, which generally resolves over time. However, women might differ in the trajectory of the distress experience. Women participating in a routine OC screening program (*n* = 373) who received an abnormal screening result completed a baseline assessment prior to a follow-up screening test to clarify the nature of their abnormal result. All women were subsequently informed that no malignancy was present, and follow-up assessments were completed one and four months post-baseline. Demographic, clinical, dispositional (optimism, monitoring), and social environmental (social constraint, social support) variables were assessed at baseline. OC-specific distress was assessed at all three assessments. Trajectory analyses identified three distress trajectories differing in the baseline level of distress. A high decreasing trajectory, representing about 25% of women, was characterized by high levels of distress at baseline with distress declining over time, but still elevated at four-month follow-up. In contrast, a no distress trajectory group, representing about 30% of women, was characterized by essentially no distress at any time point. Principal risk factors for membership in the high decreasing trajectory group included a family history of OC, lower dispositional optimism, and greater social constraint. These risk factors could be used to target resources efficiently towards managing women at risk for potentially clinically-significant distress after receipt of an FP OC screening test.

## 1. Introduction

Participation in cancer screening can foster early detection and treatment and thus improved prognosis for some cancers. However, participation in cancer screening is not without its drawbacks. All cancer screening tests yield some proportion of inconclusive or abnormal results. Such results typically require additional follow-up to determine if malignancy is present. Follow-up might involve repeat of the original screening test, additional imaging studies, diagnostic laparoscopy, or surgery. The majority of the time, follow-up reveals that malignancy is not present, and this constitutes a false positive (FP) screening test, which may not be psychologically benign. A survey of recipients of FP results in the course of breast, prostate, cervical, or colorectal cancer screening found that 40% described their experience as “very scary” or “the scariest time of my life” [1].

Ovarian cancer (OC) is associated with an excellent prognosis when diagnosed and treated early. The five-year survival rate for women diagnosed with localized disease is 92% [2]. However, the majority of women with OC (59%) are diagnosed with late stage disease where the overall five-year survival rate is much lower (29%). Consequently, considerable effort has been expended to develop cost-effective approaches to screening for OC in symptomatic, average risk women [3,4,5,6,7,8]. As no approach has been shown to reduce OC-specific mortality in a prospective, randomized trial, the U.S. Preventive Services Task Force recommends against routine screening for OC in asymptomatic, average risk women [9]. In contrast, screening for OC is widely used for asymptomatic women at elevated risk for OC. A physician survey found 33% believed transvaginal sonography and CA125 testing were effective screening tests for OC, and 28% and 65% would offer OC screening to low- and medium-risk women, respectively [10].

For asymptomatic women at average risk for OC, 5–10% of OC screening tests yield an inconclusive or abnormal result, requiring follow-up [11]. The vast majority of these inconclusive or abnormal results are FP results: follow-up indicates no malignancy present. However, receipt of an FP result may not be psychologically benign. A review of research examining the psychological impact of an FP OC screening test result concluded that FP test results are associated with an increase in OC-specific distress [12]. This distress appears to decline over time, but remains elevated relative to pre-screening levels even several months after follow-up has ruled out malignancy [12,13].

This general pattern of distress following an FP test result may not describe the trajectory of distress evidenced by any individual woman. Some women might demonstrate no increase in distress following an FP result, while other women might have a pronounced and persistent increase in distress. Clinically, it is important to recognize the potential for different distress trajectories. Trajectory analyses have been conducted with respect to distress [14], depressive symptoms [15,16], fatigue [17], physical activity [18], and sleep disturbance [19] after breast cancer diagnosis and treatment and anxiety and depression after coronary artery bypass surgery [20]. However, no research has examined whether distinct distress trajectories are evident following an FP screening test result in the OC setting, or any cancer screening setting for that matter. 

In addition to identifying distinct distress trajectories, it is important to identify “risk” factors associated with particular trajectories. Identification of characteristics associated with trajectories characterized by severe or persistent distress can facilitate efforts to prevent or minimize distress following an FP screening test result. Several studies have identified characteristics associated with greater distress following an FP OC screening test [21,22,23,24,25]. These include less education [25], a family history of OC [21], no previous FP test result [25], a less extensive OC screening history [25], a monitoring information coping style [21,23,24], low dispositional optimism [21,25], and greater social constraint [21,25]. Greater distress has also been associated with the combination of a monitoring coping style and family history of OC [22,25]. While suggestive of characteristics that might serve as “risk factors” for greater distress in trajectory analyses, all these studies identified predictors of greater distress at a specific point in time. None of these studies identified characteristics associated with different trajectories of distress following an FP OC screening test result. 

The purpose of this study is twofold. First, we identify different trajectories of cancer-specific distress following receipt of an FP OC screening result during routine OC screening. Second, we identify demographic, clinical, dispositional, and social-environmental characteristics associated with different trajectories. No previous studies have examined the trajectories of OC-specific distress in the OC screening setting. However, based on trajectory analyses of distress in other settings [15,16,20], we expect trajectories to identify several groups of women differing with respect to baseline distress levels with distress generally diminishing over time [12]. Based on previous research [21,23,24,25], we expect women evidencing trajectories involving more severe and persistent distress to be characterized by less education, greater social constraint, less optimism, a monitoring coping style, a family history of OC, no history of a previous FP screening test result, and a less extensive prior history of OC screening.

## 2. Materials and Methods

### 2.1. Sample

Study participants were in the University of Kentucky Ovarian Cancer Screening Program [26]. This program offers free, annual transvaginal sonography (TVS) screening to asymptomatic women ≥ 50 years of age and asymptomatic women 25–49 years of age with at least one first degree relative with OC. Women < 50 years of age with a personal history of breast cancer also qualify for inclusion. Women receiving an abnormal TVS screening test result during the course of routine, annual screening are typically asked to return within 3–12 weeks for a repeat TVS test.

### 2.2. Procedure

Women who received an abnormal TVS screening test result and were scheduled to return for a follow-up TVS screening test were identified from clinical records. Prior to their follow-up TVS test, women met with research staff, the study was described, and written informed consent was obtained from all participants. Less than 5% of eligible women invited to participate declined. Ethical approval for this study was obtained from the University of Kentucky Institutional Review Board.

All women completed a baseline assessment immediately prior to their follow-up TVS screening test. Follow-up telephone assessments were completed 1 month and 4 months after baseline assessment. All women were notified sometime after the baseline assessment, but prior to the 1-month follow-up assessment results from their follow-up TVS screening test that revealed no malignancy was present.

All study procedures were conducted in accordance with the ethical principles embodied in the Declaration of Helsinki. Ethical approval for this study (Protocol #99-50127) was initially granted on 12 April 1999 by the University of Kentucky Institutional Review Board. 

### 2.3. Measures

Demographic and clinical information, dispositional characteristics, social environmental characteristics, and physical functioning were assessed at baseline. OC-specific distress was assessed at baseline and the 1- and 4-month follow-up assessments. 

### 2.4. Demographic and Clinical Information

Demographic and clinical information was assessed by self-report included age, race/ethnicity, partner status, education, and family history of OC. Family history of OC (yes vs. no) was based on whether a woman had at least one first-degree relative (FDR) with OC (mother, sister, or daughter). Clinical information collected from clinic records included the number of previous TVS screening tests and a history of an abnormal TVS screening test result prior to the most recent abnormal TVS test result (yes vs. no).

### 2.5. Dispositional Characteristics

Dispositional optimism was assessed using the Life Orientation Test-Revised (LOT-R) [27]. The LOT-R is a 10-item measure of dispositional optimism and yields a total optimism score. Informational coping style was measured using the Miller Behavioral Styles Scale-Short Form (MBSS-SF) [28]. The MBSS-SF consists of 2 stressor scenarios followed by 8 statements representing coping strategies potentially implemented in response to that stressor. separate monitoring and blunting scores can be computed. 

### 2.6. Social Environmental Characteristics

Social support was assessed using the Duke-UNC Functional Social Support Questionnaire (Duke-SSQ) [29]. The Duke-SSQ is an 8-item measure of functional social support yielding a total social support score. Social constraint was assessed using the 15-item “Friends and Family” version of the Social Constraint Scale (SCS) [30]. The SCS measures the extent an individual’s social environment inhibits the expression of thoughts and feelings about a stressful event; in this case, “your experience with ovarian cancer screening.” A total social constraint score was calculated.

### 2.7. Physical Functioning

Physical functioning was assessed using the Medical Outcomes Study 12-Item Short Form Health Survey (SF-12) [31]. The SF-12 is a 12-item measure of physical and mental health status. The physical functioning subscale score was used in subsequent analyses.

### 2.8. OC-Specific Distress

OC-specific distress was assessed by the Impact of Events Scale (IES) [32]. The IES is a commonly-used 15-item measure of current distress associated with a specific potential stressor. In this study, women completed the IES with regard to the specific potential stressor “the possibility that you will develop ovarian cancer in your lifetime.” Each of the 15 IES items represents a particular symptom that might occur in response to a specific potential stressor. For example, “I thought about it when I didn’t mean to” or “I stayed away from reminders about it.” For each of the 15 IES items, women rated the frequency with which that symptom occurred in the last 7 days on a 4-point scale with response options ranging from “not at all” to “often.” The IES yields subscale scores for intrusion (7 items; score range 1–35) and avoidance (8 items; score range 1–40). IES-Intrusion subscale scores represent the extent to which a woman’s response to the specific potential stressor is characterized by frequent unwanted reminders of the stressor and an inability to control one’s thoughts about the stressor. IES-avoidance subscale scores represent the extent to which a woman’s response to the specific potential stressor is characterized by frequent purposive attempts to avoid thoughts or reminders about the stressor. 

### 2.9. Statistical Analyses

Two group-based trajectory models [33] were fit to identify trajectories of OC-specific distress; one for IES-avoidance and one for IES-intrusion scores. The underlying distribution was assumed to follow a zero inflated Poisson distribution to account for the excess presence of zeros in IES subscale scores. The number of groups and shape of each trajectory were chosen based on the Bayesian information criterion (BIC), as well as the restriction that each identified trajectory should include at least 20% of study participants. Additionally, we aimed to maximize the average of the posterior probabilities of group membership for individuals assigned to each group. The final model was chosen such that trajectories were distinct and interpretable. 

After specification of group number and trajectory shapes, time-invariant covariates were added to the model to examine the association of demographic, clinical, dispositional, and social environmental characteristics with trajectory group membership. Predictors of distress trajectories from the multivariate model were presented as estimated odds-like ratios (OLR). All analyses were performed using SAS, Version 9.4 (Cary, NC, USA), with an alpha level of 0.05.

## 3. Results

The final sample included 373 women receiving an FP TVS screening test result during participation in routine TVS screening. 

**Figure 1:**
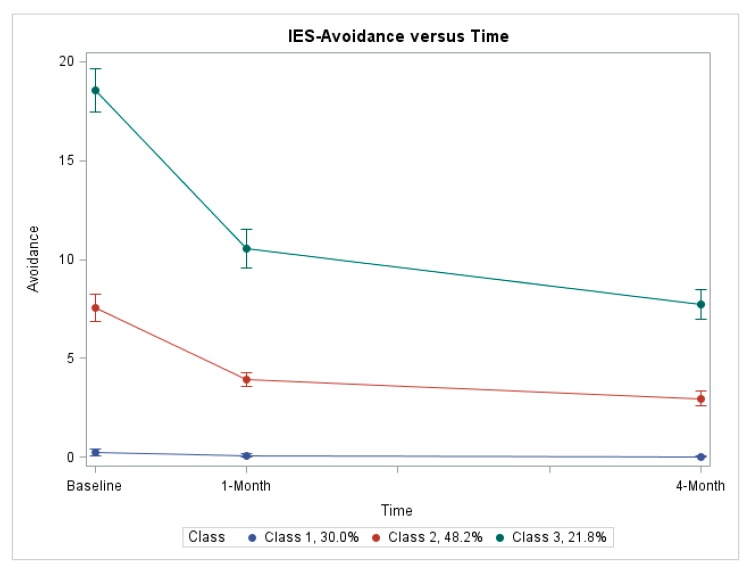
Estimated trajectories of avoidant thoughts as measured by the Impact of Events Scale (IES) instrument. Estimated trajectories of ovarian cancer (OC)-specific distress as measured by the IES-avoidance subscale in group-based trajectory modeling (*n* = 373). Class membership based on posterior probabilities from the unconditional model. The solid lines represent the fitted means and 95% confidence intervals, while the dotted lines represent the actual means at each assessment.

**Figure 2:**
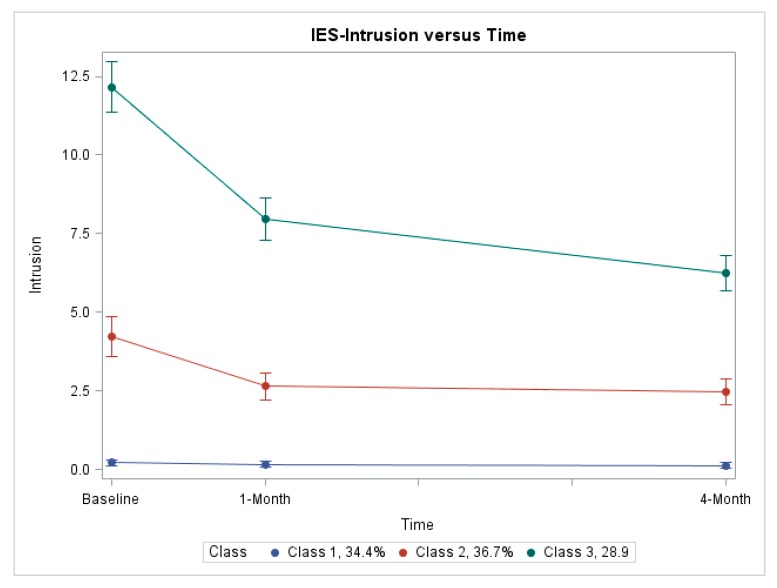
Estimated trajectories of intrusive thoughts as measured by the IES instrument. Estimated trajectories of OC-specific distress as measured by the IES-intrusion subscale in group-based trajectory modeling (*n* = 373). Class membership based on posterior probabilities from the unconditional model. The solid lines represent the fitted means and 95% confidence intervals, while the dotted lines represent the actual means at each assessment.

Figure 1 and Figure 2 represent the estimated trajectories for IES-avoidance and IES-intrusion subscale scores. For both subscales, three distinct trajectories of similar shape were identified: Class 1, no distress; Class 2, medium decreasing distress; and Class 3, high decreasing distress. For the IES-avoidance model, the proportion of study participants evidencing each of the three trajectories (i.e., class proportions) were 30.0%, 48.2%, and 21.8%, respectively (Figure 1). For the IES-intrusion model, the class proportions were 34.4%, 36.7%, and 28.9%, respectively (Figure 2). For the IES-avoidance model, mean group membership probabilities over subjects were 94%, 96%, and 95% for Class 1, Class 2, and Class 3, respectively. For the IES-intrusion model, mean probabilities were 94%, 93%, and 94% for Class 1, Class 2, and Class 3, respectively. 

To identify predictors associated with the probabilities of membership in various trajectories, both models were re-estimated with the inclusion of time-invariant covariates. Descriptive statistics of covariates by class membership are displayed in Table 1. The average age of women in the total sample was 56.7 years (SD = 11.7), and they were highly educated overall, with years of education beyond high school. On average, women had approximately two previous routine TVS tests in the program, and the majority had no previous history of an abnormal test result. Family history of OC in an FDR was present in less than a quarter of women in the ‘no distress’ class, with higher rates reported among those in the ‘medium decreasing’ and ‘high decreasing’ classes. Of the dispositional and social environmental characteristics, optimism was slightly higher among those in the ‘no distress’ trajectory, while social constraint was highest among those in the ‘high decreasing’ class. 

Table 2 shows the results of the covariate-adjusted models including estimated odds-like ratios (OLR) and accompanying 95% confidence intervals. For continuous covariates, the Class 2 versus Class 1 estimated OLR represents the ratio of the estimated probability of group membership in Class 2 to Class 1 for every unit increase in the covariate, controlling for all other factors. For categorical covariates, the Class 2 versus Class 1 OLR represents the ratio of the estimated probability of group membership in Class 2 to Class 1 at the first level of the covariate versus the second level of the covariate, controlling for all other factors. 

Three variables were associated with increased likelihood of membership in Class 2 (medium decreasing) versus Class 1 (no distress) for both IES-intrusion and IES-avoidance scores (Table 2): a family history of OC in an FDR (IES-avoidance OLR = 2.53; *p* < 0.01; IES-intrusion OLR = 2.91; *p* < 0.01), no history of an abnormal TVS test result (IES-avoidance OLR = 2.26; *p* < 0.05; IES-intrusion OLR = 2.60; *p* < 0.05), and greater social constraint (IES-avoidance OLR = 1.16; *p* < 0.001; IES-intrusion OLR = 1.15; *p* < 0.001). Women with a family history of OC, no prior abnormal TVS test result, and reporting greater social constraint were more likely to belong to the medium decreasing distress trajectory than the no distress trajectory for both IES subscales. Additionally, better physical functioning (OLR = 1.01; *p* < 0.05) and lower optimism (OLR = 0.91; *p* < 0.05) were associated with greater likelihood of membership in Class 2 vs. Class 1 for IES-intrusion scores, while a greater monitoring coping style was associated with a greater likelihood of membership in Class 2 vs. Class 1 for IES-avoidance scores (OLR = 1.24; *p* < 0.05).

Three variables were associated with an increased likelihood of membership in Class 3 (high decreasing) versus class 1 (no distress) for both IES-intrusion and IES-avoidance scores (Table 2): a family history of OC in an FDR (IES-avoidance OLR = 2.55; *p* < 0.05; IES-intrusion OLR = 5.41; *p* < 0.01), lower dispositional optimism (IES-avoidance OLR = 0.89; *p* < 0.05; IES-intrusion OLR = 0.77; *p* < 0.001), and greater social constraint (IES-avoidance OLR = 1.29; *p* < 0.001; IES-intrusion OLR = 1.27; *p* < 0.001). Women with a family history of OC, lower dispositional optimism, and reporting greater social constraint were more likely to belong to the high decreasing distress trajectory than the no distress trajectory for both IES subscales. Additionally, lower education (OLR = 0.85; *p* < 0.05), a monitoring informational coping style (OLR = 1.39; *p* < 0.05), and greater social support (OLR = 1.12; *p* < 0.05) were associated with a greater likelihood of membership in Class 3 vs. Class 1 for IES-intrusion scores only.

Finally, only a single variable was associated with an increased likelihood of membership in Class 3 (high decreasing) vs. Class 2 (medium decreasing) for both IES-intrusion and IES-avoidance scores: greater social constraint (IES-avoidance OLR = 1.11; *p* < 0.001; IES-intrusion OLR = 1.11; *p* < 0.001). Additionally, fewer previous routine TVS tests (OLR = 0.91; *p* < 0.05), lower dispositional optimism (OLR = 0.84; *p* < 0.001), and greater social support (OLR = 1.10; *p* < 0.05) were associated with an increased likelihood of membership in Class 3 vs. Class 2 for IES-intrusion scores. 

## 4. Discussion

In general, results matched our expectations regarding trajectory shapes and characteristics associated with trajectory group membership. Estimated trajectories were similar for both IES-intrusion and IES-avoidance models. For both models, three distinct, non-overlapping trajectories were identified, each differing with regard to the baseline level of OC-specific distress. 

For both IES subscales, a trajectory characterized by essentially no reported distress at baseline and through four-month follow-up was identified (i.e., no distress, Class 1). This trajectory was evidenced by about a third of women for both subscales. From a clinical standpoint, women in this trajectory group appear undisturbed by their FP screening test result and would not appear to require any additional attention. 

In contrast, for both IES subscales, a trajectory characterized by high reported distress at baseline was identified (i.e., high decreasing, Class 3). While distress declined over time, distress still remained at about 50% of the baseline level four months after baseline. This trajectory was evidenced by about 25% of all women for both IES subscales; not a trivial proportion of women. On average, women in this high decreasing trajectory group reported baseline IES-avoidance and IES-intrusion scores of approximately 19 and 12, respectively, yielding an IES-total score of about 31. As IES-total scores > 19 are considered “high” [34], approximately one in four women report what is considered clinically-significant distress at the time of their follow-up TVS screening test. Distress was less at the one-month follow-up assessment, no doubt due to being informed that follow-up TVS testing indicated no malignancy was present. However, IES-total scores at both 1- and 4-month follow-up remained in the range of 18 and 16, respectively, only slightly below the threshold for “high” distress. Women in this trajectory group may require additional clinical attention to manage distress after their FP screening test. 

For both IES subscales, a third, intermediate trajectory was identified (i.e., medium decreasing; Class 2). This trajectory was evidenced by 38% and 47% of women for IES-intrusion and IES-avoidance scores, respectively. On average, women in this trajectory group reported baseline IES-intrusion and -avoidance scores of about 4.5 and 7.5, respectively, thus presenting an IES-total score at baseline of about 12; a “medium” distress level [34]. Distress scores declined after baseline, with IES-total scores of about 6.5 at four-month follow-up, on average, representing “low” distress [34]. Clinically, this group of women might merit some attention at baseline, though clearly, clinical priority should be given to women in the high decreasing (i.e., Class 3) trajectory group. 

Clinical efforts to manage distress in the 25% of women demonstrating the high decreasing distress trajectory are enhanced by the identification of risk factors associated with this trajectory group. These included family history of OC, lower dispositional optimism, greater social constraint, a monitoring coping style, and greater social support. All these factors had previously emerged as cross-sectional predictors of distress following an FP OC screening test result [21,22,23,24,25]. Awareness that OC runs in one’s family, a monitoring coping style [35,36], and a weaker tendency to adopt an optimistic stance in interpreting threatening events [37,38] would all be expected to amplify the threat experienced in response to an FP OC screening test result. The experience of greater social constraint in response to efforts to express thoughts and feelings regarding a woman’s experience with OC screening could stymie coping with the threat posed by an FP test result, resulting in greater distress [39,40]. An unexpected finding was the association of greater social support with the high decreasing distress trajectory group. This relationship was only for IES-intrusion scores. Greater social support might foster emotional and cognitive processing of a threatening event, in this case an FP screening test result [39,41]. Consequently, higher intrusion scores then might be a marker for greater cognitive processing rather than an indicator of distress [41,42]. 

Finally, the absence of prior experience with an abnormal TVS test result was associated with the medium decreasing distress trajectory. At least some distress is likely to result the first time a woman experiences an abnormal test result during routine OC screening. Some women experience multiple abnormal screening test results in the course of undergoing routine screening over their lifetime. Having experienced an abnormal TVS test result previously and presumably having undergone follow-up testing that determined the absence of malignancy resulted in reducing the threat perceived by any subsequent abnormal OC screening test. 

Study limitations should be noted. The sample was mostly Caucasian and limited to a single site. Consequently, caution is advised in generalizing results to minority women or the total population. Generalization of results to screening for other cancers, particularly cancers involving men, may also be limited. Finally, the baseline assessment was not administered until immediately prior to a follow-up TVS screening test to clarify the nature of an earlier abnormal TVS test result occurring 3–12 weeks earlier. Consequently, this study did not characterize the complete trajectory of distress. Women in our no distress trajectory group may have experienced distress immediately after learning of their abnormal test result, which could then dissipate over the 3–12 weeks leading up to the baseline assessment in the present study. Similarly, baseline distress evident in women in the high decreasing and medium decreasing trajectory groups may not represent distress experienced immediately following their abnormal test result. Distress may have been greater or lesser than reported at baseline upon learning of their abnormal test result. 

## 5. Conclusions

Previous research suggested an FP OC screening test result is associated generally with an increase in distress followed by a gradual decline in distress [12,13]. The present study, however, suggests this general conclusion obscures the existence of distinct distress trajectories. Importantly, these different trajectories have different implications for clinical management of distress in response to FP screening test results. About one in three women report essentially no distress following an FP test result and thus require no particular clinical attention. In contrast, about one in four women report a high level of distress following an FP test result. While distress declines over time, it remains relatively high even several months after malignancy has been ruled out. These women, characterized primarily by a family history of OC, less dispositional optimism, and greater social constraint, could benefit from clinical intervention to manage their distress. Clinical efforts could be targeted efficiently toward women evidencing this cluster of risk factors.

## Figures and Tables

**Table 1:** Distribution of demographic, clinical, dispositional, and social environmental characteristics by estimated trajectory (*n* = 373). TVS, transvaginal sonography; FDR, first-degree relative.

**Avoidance**			
**Covariates**	**Class 1**	**Class 2**	**Class 3**
	**No Distress**	**Medium Decreasing**	**High Decreasing**
	***n* = 118**	***n* = 181**	***n* = 74**
	**Mean (SD), Median (IQR) or %**	**Mean (SD), Median (IQR) or %**	**Mean (SD), Median (IQR) or %**
Age	58.3 (11.7)	56.1 (12.2)	55.7 (10.3)
Years of education	14.2 (2.9)	14.0 (2.7)	13.8 (2.9)
Number of previous routines TVS tests	2.5 (1.0–8.0)	2.0 (1.0–8.0)	1.5 (0.0–5.0)
No history of abnormal TVS test result	72.9	82.3	82.4
Family history of OC in an FDR	22.0	36.5	32.4
Physical functioning	79.0 (28.0)	78.7 (29.5)	77.0 (27.7)
Optimism	17.6 (3.4)	16.3 (3.7)	15.1 (3.7)
Monitoring	3.3 (1.6)	4.0 (1.7)	3.9 (1.8)
Social support	38.0 (34.0–40.0)	36.0 (31.0–39.0)	33.0 (28.5. 37.0)
Social constraint	16.0 (15.0–18.0)	19.0 (15.5–25.0)	27.0 (20.5, 34.5)
**Intrusion**			
**Covariates**	**Class 1**	**Class 2**	**Class 3**
	**No Distress**	**Medium Decreasing**	**High Decreasing**
	***n* = 143**	***n* = 140**	***n* = 90**
	**Mean (SD), Median (IQR) or %**	**Mean (SD), Median (IQR) or %**	**Mean (SD), Median (IQR) or %**
Age	57.7 (11.6)	57.9 (10.8)	53.3 (12.6)
Years of education	14.5 (2.9)	13.9 (2.7)	13.6 (2.8)
Number of previous routines TVS tests	2.0 (1.0–8.0)	2.0 (0.0–7.5)	1.0 (0.0–4.0)
No history of abnormal TVS test result	72.0	83.6	84.4
Family history of OC in an FDR	22.4	32.9	42.2
Physical functioning	78.0 (29.4)	80.9 (26.9)	75.6 (30.2)
Optimism	17.7 (3.3)	16.6 (3.3)	14.3 (4.0)
Monitoring	3.3 (4.2)	3.7 (1.7)	4.3 (1.9)
Social support	38.0 (33.0–40.0)	36.0 (30.0–39.0)	36.0 (31.0–39.0)
Social constraint	16.0 (15.0–19.0)	20.0 (15.0–26.0)	25.5 (19.0–32.0)

**Table 2:** Multivariate association between demographic, clinical, dispositional, and social environmental characteristics and OC-specific distress trajectory membership (*n* = 373).

	Medium Decreasing Versus No Distress	High Decreasing Versus No Distress	High Decreasing Versus Medium Decreasing
	(Class 2 Versus Class 1)	(Class 3 Versus Class 1)	(Class 3 versus Class 2)
	Estimated OLR (95% CI)	Estimated OLR (95% CI)	Estimated OLR (95% CI)
**Age ^a^**			
**Avoidance**	1.03 (0.78, 1.34)	1.08 (0.75, 1.54)	1.05 (0.75, 1.27)
**Intrusion**	1.24 (0.94, 1.63)	1.00 (0.70, 1.42)	0.81 (0.59, 1.10)
**Years of education**			
**Avoidance**	0.96 (0.87, 1.06)	0.93 (0.81, 1.06)	0.97 (0.87, 1.08)
**Intrusion**	0.91 (0.83, 1.01)	0.85 (0.75, 0.97) *	0.94 (0.83, 1.05)
**Number of previous routines TVS tests**			
**Avoidance**	1.01 (0.95, 1.07)	0.97 (0.89, 1.06)	0.96 (0.89, 1.04)
**Intrusion**	1.00 (0.94, 1.06)	0.91 (0.83, 1.01)	0.91 (0.84, 0.99) *
**No history of abnormal TVS test result**			
**Avoidance**	2.26 (1.08, 4.75) *	2.05 (0.74, 5.64)	0.90 (0.38, 2.17)
**Intrusion**	2.60 (1.23, 5.52) *	2.18 (0.78, 6.10)	0.83 (0.33, 2.14)
**Family history of OC in an FDR**			
**Avoidance**	2.53 (1.34, 4.81) **	2.55 (1.10, 5.92) *	1.01 (0.51, 1.98)
**Intrusion**	2.91 (1.52, 5.60) *	5.41 (2.39, 12.26) **	1.86 (0.93, 3.70)
**Physical functioning**			
**Avoidance**	1.01 (0.99, 1.02)	1.01 (0.99, 1.03)	1.01 (0.99, 1.02)
**Intrusion**	1.01 (1.00, 1.03) *	1.01 (0.99, 1.02)	0.99 (0.98, 1.01)
**Optimism**			
**Avoidance**	0.95 (0.87, 1.03)	0.89 (0.80, 0.99) *	0.93 (0.86, 1.02)
**Intrusion**	0.91 (0.84, 0.99) *	0.77 (0.69, 0.86) ***	0.84 (0.77, 0.92) ***
**Monitoring**			
**Avoidance**	1.24 (1.05, 1.46) *	1.18 (0.96, 1.46)	0.96 (0.80, 1.14)
**Intrusion**	1.18 (0.99, 1.39)	1.39 (1.13, 1.70) *	1.18 (0.99, 1.40)
**Social support**			
**Avoidance**	0.98 (0.93, 1.03)	1.00 (0.93, 1.07)	1.02 (0.97, 1.08)
**Intrusion**	1.02 (0.97, 1.07)	1.12 (1.05, 1.20) *	1.10 (1.04, 1.17) *
**Social constraint**			
**Avoidance**	1.16 (1.09, 1.24) ***	1.29 (1.21, 1.39) ***	1.11 (1.07, 1.16) ***
**Intrusion**	1.15 (1.09, 1.21) ***	1.27 (1.19, 1.36) ***	1.11 (1.06, 1.16) ***

* *p* < 0.05, ** *p* < 0.01, *** *p* <0.001. ^a^ Corresponds to a 10-unit increase in age.
